# TextNetTopics Pro, a topic model-based text classification for short text by integration of semantic and document-topic distribution information

**DOI:** 10.3389/fgene.2023.1243874

**Published:** 2023-10-05

**Authors:** Daniel Voskergian, Burcu Bakir-Gungor, Malik Yousef

**Affiliations:** ^1^ Computer Engineering Department, Faculty of Engineering, Al-Quds University, Jerusalem, Palestine; ^2^ Department of Computer Engineering, Faculty of Engineering, Abdullah Gul University, Kayseri, Türkiye; ^3^ Department of Information Systems, Zefat Academic College, Zefat, Israel; ^4^ Galilee Digital Health Research Center, Zefat Academic College, Zefat, Israel

**Keywords:** text classification, feature selection, topic selection, topic projection, topic modeling, short text, sparse data

## Abstract

With the exponential growth in the daily publication of scientific articles, automatic classification and categorization can assist in assigning articles to a predefined category. Article titles are concise descriptions of the articles’ content with valuable information that can be useful in document classification and categorization. However, shortness, data sparseness, limited word occurrences, and the inadequate contextual information of scientific document titles hinder the direct application of conventional text mining and machine learning algorithms on these short texts, making their classification a challenging task. This study firstly explores the performance of our earlier study, TextNetTopics on the short text. Secondly, here we propose an advanced version called *TextNetTopics Pro*, which is a novel short-text classification framework that utilizes a promising combination of lexical features organized in topics of words and topic distribution extracted by a topic model to alleviate the data-sparseness problem when classifying short texts. We evaluate our proposed approach using nine state-of-the-art short-text topic models on two publicly available datasets of scientific article titles as short-text documents. The first dataset is related to the Biomedical field, and the other one is related to Computer Science publications. Additionally, we comparatively evaluate the predictive performance of the models generated with and without using the abstracts. Finally, we demonstrate the robustness and effectiveness of the proposed approach in handling the imbalanced data, particularly in the classification of Drug-Induced Liver Injury articles as part of the CAMDA challenge. Taking advantage of the semantic information detected by topic models proved to be a reliable way to improve the overall performance of ML classifiers.

## 1 Introduction

Numerous scientific research papers are being published daily with the increasing advancement of computer and information technologies and digital platforms. Publication output is reported to reach 2.6 million worldwide in 2018 (White, 2019). 36% of publications correspond to health-related research (i.e., health sciences, biological and biomedical sciences) as the largest global field of science, followed by other domains such as computer and information sciences. Manual classification of these documents is tedious and prone to human mistakes or deletions due to the immense size of textual data. Therefore, to deal with such documents efficiently, there is a need for automatic document classification (ATC) to classify articles accurately and quickly into one or more predefined categories according to their content (e.g., one topic or another). The ATC process will assist in organizing documents and facilitate the extraction of relevant information related to the topic of interest. Most of the studies in this field considered the abstract section or combined the abstract with the title for classification. Only a few studies in this field used the titles by itself. Article titles are brief word descriptions of the articles’ content with valuable information that one can utilize in document classification. However, due to the concise nature of article titles, title-based article classification presents a significant challenge, as it can be difficult to identify key features that are distinctive enough.

In fact, the number of features in a short text is a small portion of the set of all features presented in all of the training records. This situation creates feature sparseness. Although this is problematic for regular-sized text, it is more critical for short text. In particular, short texts tend to have diversification in content; the same topic can be expressed in multiple ways, which increases the feature space and reduces the frequency of a feature occurring in a given record, as well as the number of records in which a particular feature occurs, leading to the scarcity of feature overlap. This situation makes it challenging to accurately determine a feature’s salience in a specific class in supervised machine-learning tasks (Bollegala et al., 2018).

As mentioned above, the severe data sparsity, high dimensionality, lack of word co-occurrence, and insufficient shared context are other distinctive characteristics that distinguish short texts from general ones. Inevitably this situation negatively affects the classification performance when conventional machine-learning methods are used. In other words, traditional classification methods are not optimized for use in such a context (Alsmadi and Gan, 2019; Song et al., 2014).

*Topic Modeling* (TM) is an unsupervised learning task aiming to discover latent thematic contents in a collection of text documents. TM provides a simple way to analyze, understand, summarize, and categorize large volumes of unlabeled text (Kherwa and Bansal, 2018). The basic notion of the classical topic models assumes that a document is generated from a multinomial distribution over topics, whereas a topic has a multinomial distribution over terms. Once estimated (e.g., using Gibbs sampling), a topic model provides two byproducts: 1) a *document-topic* distribution matrix containing the distribution of topics over documents (reflecting a high-level representation of the document’s semantics). 2) a *topic-word* distribution matrix containing the distribution of words across topics (i.e., TM assigns a word to a topic with a probability). Each topic comprises a group of words that co-occur in documents according to specific patterns. In other words, a topic model can differentiate words with distinct semantics and group them into topics based on their co-occurrence (Vayansky and Kumar, 2020; Barde and Bainwad, 2017). In addition to discovering topics and uncovering the latent semantics of the unstructured text collection, research studies used TM with broad success for text classification tasks (Xia et al., 2019).

In this aspect, TextNetTopic (Yousef and Voskergian, 2022) is a novel text classification approach that performs feature selection by selecting top-ranked topics (a topic is a group of terms detected by a Topic Model) as features to train the classifier. It fulfills dimensionality reduction while preserving more thematic and semantic information in the text document representations. Typically, conventional methods for feature selection involve evaluating the importance or significance of individual words by assigning scores to each one without considering the relationships between words. In contrast, the TextNetTopics approach relies on the fact that words are related and should be organized into topics that are detected by using a suitable topic modeling technique. TextNetTopics was developed to perform topic selection rather than word selection.

This study compares the performance of TextNetTopics (Yousef and Voskergian, 2022) on short text with other competitive feature selection algorithms, such as Extreme Gradient Boosting (XGBoost) (Chen and Guestrin, 2016), Fast Correlation Based Filter for Feature Selection (FCBF) (Senliol et al., 2008), and Select K Best (SKB) (Pedregosa et al., 2011). In addition, this article proposes a novel short text classification approach called TextNetTopics Pro [an enhancement of TextNetTopics (Yousef and Voskergian, 2022)], utilizing various short text topic models designed for sparse data, such as the Gibbs Sampling algorithm for Dirichlet Multinomial Mixture (GSDMM) (Yin and Wang, 2014), Gamma-Poisson Mixture (GPM) (Mazarura et al., 2020), Biterm Topic Model (BTM) (Yan et al., 2013), Word Network Topic Model (WNTM) (Zuo et al., 2016a), Self-Aggregation-based Topic Model (SATM) (Quan et al., 2015), Pseudo-document-based Topic Model (PTM) (Zuo et al., 2016b), Latent feature model with DMM (LFDMM) (Nguyen et al., 2015), General Pólya Urn Dirichlet Multinomial Mixture (GPU-DMM), and General Pólya Urn Poisson-based Dirichlet Multinomial Mixture (GPU-PDMM) (Li et al., 2018).

This research study is primarily driven by a fundamental research question: What is the extent of the impact when employing a novel scheme that combines topics as lexical and semantic features on text classification, as compared to traditional methods that rely exclusively on a single feature type?

We organize the rest of this article as follows. Section 2 provides an overview of related research utilizing topic models in short-text classification. Section 3 describes different topic models for short text. Section 4 presents a brief overview of TextNetTopics and describes our proposed approach for short-text classification. Section 5 elaborates on the experimental setup and evaluation. Section 6 encompasses the results and discussions. In section 7, we draw our conclusion and future work.

## 2 Related work—Short text classification approaches utilizing topic models

The short text problem was studied in many areas, such as social media (Al Qundus et al., 2020). Various studies proposed short-text classification approaches utilizing topic modeling to tackle and alleviate the issue of data sparsity in short-text classification. They utilized two approaches: 1) enriching short texts with external domain knowledge base resources (external resource-based approach). In other words, topics extracted from large-scale external corpora are added into short text documents as external features. 2) expanding short texts using internal knowledge acquisition (corpus-based approach). The identified topics from the same short-text corpus can be seen as features. For instance, the distribution of the topics across documents can be used to generate compact and dense document representations in low-dimensional semantic space for text classification (Sun and Chen, 2018; Qiang et al., 2022).

Using the first approach, Vo and Ock (2015) proposed a framework for short-text classification. They estimated a Latent Dirichlet Allocation (LDA)-based topic model through Gibbs sampling on three universal datasets (DBLP, LNCS, and Wikipedia). Then, they used the topic model to improve features in short text documents by merging the optimal number of matching topics (according to predefined equations) to each word and combining adapted topics’ words as external features. Finally, they used the enriched data to construct a feature vector before applying it to various classification algorithms.

Zhang and Zhong (2016) proposed a short-text classification framework consisting of three phases (topic learning, topic/word vector learning, and classification). They used a corpus related to the short text to be classified to build a topic model using LDA. Then, they enriched the corpus and short texts by assigning topics to words (word-topic assignment) and integrating them into the text (they treated topics as new words). Then the enriched corpus is used to learn both word and topic vector representations interactively via modified Continuous Bag of Words (CBOW) and skip-gram methods. Finally, they represent the features of enriched short texts (i.e., words and topics) by the learned vectors for training the classifier.

Like Zhang’s work, Sun et al. (2022) proposed an enhanced approach for acquiring vector representations for topics and words. They created word-topic pairs by matching each word with its corresponding topic. The word in the pair was used to predict contextual words, while the topic was used to predict contextual topics. In other words, a topic is predicted only by other topics without words. Following this approach, they learn two sets of vector representations for the short text: word and topic vectors. In addition, they employed a supervised Multi-Cluster Feature Selection algorithm to select the optimal topic subset and proposed a novel topic-merging strategy to mitigate the loss of features (topics). Finally, short text matrices are created by utilizing learned vector representations, and these matrices are then inputted into a convolution neural network.

Yang et al. (2013) proposed a short-text classification approach by combining lexical and semantic features. They assigned each word in a short text with a learned topic from a background knowledge repository through a Gibbs sampler for LDA, then transformed the text into a semantic vector representation with a size corresponding to the number of topics, where each cell contains times of appearance of each assigned topic inside the text. Moreover, they used an improved expected cross entropy to select the top distinctive words in each category. Then, they combined those lexical features with the semantic features by mapping them to topics with different weights to reflect their discriminative characteristics.

Sun and Zhao (2017) proposed a novel feature extension approach to solving short-text classification problems. They trained the TNG algorithm, an improved topic model that can infer unigram words and phrases distribution on each topic, on an extensive text collection related to the domain as universal data. They used these features to build a feature extension library. Afterward, a Topic Weight Vector is computed for each short text, and the topic with the highest value is used to define its topic tendency. Then, appropriate candidate words and phrases associated with that topic are selected from the feature extension library to extend the original short texts. Finally, an LDA is used to obtain the document-topic distribution for the extended short texts to train an SVM classifier.

The main problem with the first approach is that it relies on a vast volume of high-quality external data, which we may lack for some special domains and languages, or it can be very costly to collect such data.

Following the second approach, Bagheri et al. (2020) introduced an ETM (enrichment by topic modeling) algorithm for clinical sentence classification. Instead of employing external knowledge repositories, ETM uses internal knowledge acquisition to enrich text by incorporating LDA’s short texts’ distribution probabilities (i.e., document-topic and topic-word probabilities), the length of the document, and the value of TF-IDF of the word to smoothen their semantic representation. They used SVM and neural network algorithms for the classification tasks.

Chen et al. (2016) proposed a novel distance metric formula for the KNN algorithm to classify short texts. It integrates the LDA semantic features with discriminative word relationship information. It considers the shared latent topics assigned to the discriminative words in the two short texts as third-party features to compare their similarity. They embedded this assumption in a modified feature vector and used the cosine method to calculate the topic similarity.

Liu et al. (2022) proposed a short text classification approach utilizing a convolutional neural network. They combined the latent document-topic vector extracted by RLDA, an enhanced LDA topic model based on the Relevance formula and the latent semantic vector representation of the document extracted by a word2vec word vector model to construct a new text feature representation; and then applied it to a four-layer CNN.

Pei et al. (2018) introduced a novel convolutional neural network, TW-CNN, employing topic information and word embedding for short text classification. The word vector matrices are first generated using LDA topic modeling and word2vec and then fed into two distinct CNNs. These CNNs consist of convolution and pooling layers and generate two different vector representations of the text. The resulting vectors are then combined with the text-topic vector acquired from LDA, which produces the final representation vector of the text. The final vector is used to perform softmax text classification.

Ge et al. (2020) classified short texts based on Word-network Triangle Topic Model (WTTM) and word vector. They aggregated the short text corpus in one document and used it to train word vectors using the Word2Vec-CBOW model. In addition, they trained WTTM based on Gibbs sampling to obtain the topic-word distribution matrix and the topic-word files. Each word inside the text is matched with the topic-word distribution matrix, selecting the topic with the highest probability and taking n words from the topic-word file as the feature extension of the original word. Finally, both vectors (word and topic extended feature vectors) are merged and used to train a random forest classifier.

As we have noticed, most studies used Latent Dirichlet location (LDA) as the main source for performing topic modeling to enrich the short text, which only works efficiently with long text. Nevertheless, many other topic modeling algorithms exist, with some of them customized for short texts and well-tuned for sparse documents, and picking a good one is not straightforward. This study proposes a novel short text classification approach called *TextNetTopics Pro,* which is an enhancement of TextNetTopics, leveraging various short text topic models, i.e., *GSDMM, GPM, BTM, WNTM, SATM, PTM, LF-DMM, GPU-DMM, and GPU-PDMM,* designed for sparse data*.* Moreover, since TextNetTopic performs feature selection, we compare its performance on short text with other competitive feature selection algorithms, such as *XGBoost*, *FCBF,* and *SKB*.

## 3 Short-text topic model

Topic modeling refers to a set of algorithms that aim to discover the underlying structures and hidden topics in an unlabeled text corpus. In contrast to regular-sized documents, inferring the latent topics and discovering the hidden semantic structure in a collection of short documents is challenging.

Insufficient word co-occurrence information within individual short texts significantly contributes to a notable decline in performance (i.e., less reliable and inferior topic inference, the resultant topics are semantically less coherent) when utilizing traditional topic models across short texts, which implicitly captures the word co-occurrence patterns at the document level to discover topics (Yan et al., 2013; Qiang et al., 2022). This situation makes classical topic models highly influenced by the length of documents and the number of documents related to each underlying topic (Zuo et al., 2016a).

Recently, researchers have proposed topic models specially designed to handle short texts to overcome the problem of shortness, severe data sparseness, high dimensionality, and the minimal availability of word co-occurrence information in each of them (Qiang et al., 2022). These studies mainly follow the following three approaches.

- Dirichlet multinomial mixture (DMM): Unlike the LDA, which adopts a complex assumption that each text is sampled over a set of topics, DMM follows a simple assumption that each text is modeled from only one latent topic.
- Global word co-occurrences: It infers latent topics leveraging the global word co-occurrence patterns obtained from the whole corpus.
- Self-aggregation: It aggregates short texts into lengthy pseudo-document before conducting topic inference (training a topic model) to improve word co-occurrence information.

Some relevant algorithms related to short-text topic modeling can be summarized as follows:

GSDMM: Yin and Wang proposed a *Gibbs Sampling algorithm for Dirichlet Multinomial Mixture*. GSDMM is inherently a mixture of a unigrams model, with the generative assumption that the document is sampled from one topic instead of multiple topics like in the LDA, and the words depend on that topic. GSDMM samples a latent topic for a document based on collapsed Gibbs sampling (Yin and Wang, 2014).

GPM: The *Gamma-Poisson Mixture* model is a topic modeling technique that employs an independent Poisson distribution to describe the frequency of word occurrences in fixed-length documents, as opposed to the GSDMM and LDA models, which use a multinomial distribution. GPM differs from the GSDMM, which uses the Dirichlet distribution as a conjugate prior to the multinomial distribution by assuming a Gamma prior distribution as a conjugate prior to the Poisson distribution. Similar to the GSDMM, the GPM model is a mixture model that assumes each document is generated from a single topic rather than a mixture of topics. The model utilizes a collapsed Gibbs sampler to automatically estimate the number of topics in a document collection (Mazarura et al., 2020).

BTM: A *Biterm Topic Model* can be considered a specialized form of the mixture of unigrams. Unlike conventional topic models that model word generation from the document level to implicitly capture word co-occurrence patterns, the BTM explicitly models the generation of biterms (unordered pairs of words co-occurring in the same context) in the whole corpus to infer topics over short text. It considers that the corpus composes a mixture of topics, and each biterm is drawn from a specific topic only (Yan et al., 2013).

WNTM: *Word Network Topic Model* is designed to tackle the sparsity and the significant imbalance in short text document distribution (i.e., heavily skewed). It can discover rare topics contained in fewer documents. WNTM learns the latent word groups (topic components) by applying the standard Gibbs sampling for LDA in a word co-occurrence network rather than the document collection. Moreover, unlike other approaches, WNTM models the distribution of latent topic components for each word instead of the distribution of topics for each document (Zuo et al., 2016a).

SATM: *Self-Aggregation-based Topic Model* integrates topic modeling and text self-aggregation (clustering) simultaneously during topic inference. It assumes that each piece of short text is sampled from a long pseudo-document hidden in the current text corpus, following the multinomial distribution. It uses the standard topic modeling (i.e., Gibbs sampling) to infer latent topics from pseudo-documents without relying on metadata or auxiliary contextual information, which is too costly for deployment and not always available. However, SATM is prone to overfitting since the number of SATM parameters grows linearly with the size of the corpus, and its time complexity is very high (Quan et al., 2015).

PTM: Like SATM, the *Pseudo-document-based Topic Model* assumes that much less normal-sized latent documents named ‘pseudo documents’ generate the vast volume of short texts in the corpus. It learns the topic distributions of pseudo documents rather than short texts. However, PTM is substantially different from SATM in its generative processes. In addition, PTM has a fixed number of parameters, so when the training corpus is in relative shortage, it gains the power to avoid overfitting (Zuo et al., 2016b).

GPU-PDMM and GPU-DMM: These algorithms are variants of DMM. Both algorithms exploit the general word semantic relations incorporated in word embeddings learned by neural network language models, such as CBOW and Glove, during the Gibbs sampling process (i.e., topic inference process) through the generalized Polya urn (GPU) model. After sampling a topic, words highly relevant to the topic are selected and linked with their semantically related words together under the same topic, even though they share limited or no co-occurrences in the modeled short-text corpus (Li et al., 2018). Regarding PDMM, the *Poisson-based Dirichlet Multinomial Mixture Model*, is a variant of DMM. Since a single-topic assumption in some short-text corpus may be too strong, it assumes a short text is sampled by one or a limited number of topics, whereas the Poisson distribution models the number of topics (Li et al., 2018).

LF-DMM: *Latent feature model with DMM* incorporates word embeddings into Dirichlet Multinomial Mixture by replacing the topic-word Dirichlet multinomial component with a two components mixture of a Dirichlet multinomial component and a latent feature (a continuous word embedding) component. It uses a binary switch variable (sampled from a Bernoulli distribution) to choose which component generates a word. The model projects the topics into the same continuous latent space as the word embeddings to estimate the word embedding component of each word. This is achieved by optimizing a regularized log-linear model (Nguyen et al., 2015).

## 4 Methodology

### 4.1 TextNetTopic

TextNetTopic is a topic model-based topic selection algorithm developed for textual data analysis that was based on a prior study TopicsRanksDC for topics ranking based on the distance between two clusters that are generated by each topic (Yousef et al., 2020). It relies on the generic Grouping, Scoring, and Modeling (G-S-M) approach (Ge et al., 2020). It uses Latent Dirichlet Allocation (LDA) as a default topic model to detect latent topics, where each topic contains the most related words indicative of the underlying topic. TextNetTopics scores the topics (a topic here represents a group of semantically related words) and finds the top significant *r* topics, determined by their high scores (i.e., mean classification accuracy), which form an aggregated subset of words that effectively discriminate the two classes of documents (in case we are dealing with a binary classification problem). These selected topics are then used to train the classifier. Scoring topics is computed by using a machine learning model (i.e., Random Forest algorithm) (Yousef and Voskergian, 2022). As shown in Yousef and Voskergian (2022), TextNetTopics outperformed other traditional feature selection techniques for regular-sized documents.

Similar bioinformatics tools were developed based on the G-S-M that also perform grouping based on prior biological knowledge. These are some of those tools: maTE (Yousef et al., 2019) which uses microRNA target gene information for grouping the genes; miRcorrNet (Yousef et al., 2021a) and miRModuleNet (Yousef et al., 2022), which detect feature sets via concurrently analyzing mRNA and miRNA expression datasets; CogNet (Yousef et al., 2021b) and PriPat (Yousef et al., 2023) that use KEGG pathway information for grouping the genes; GediNet (Qumsiyeh et al., 2022) that uses disease gene associations, miRdisNET (Jabeer et al., 2023) that uses miRNA target gene information while assigning the genes into sets, GeNetOntology (Ersoz et al., 2023) uses the Ontology information for grouping the genes. 3Mint (Unlu Yazici et al., 2023) is a recent tool that integrates 3-omics datasets in order to detect groups and apply the G-S-M model.

### 4.2 TextNetTopics Pro

TextNetTopics Pro is an enhancement over TextNetTopics. It is a topic model-based approach to short-text classification, which integrates lexical information (topic words) and document-topic distribution information. In other words, it strives to find the top-ranked *r* topics, each defined as sets of semantically related words, that align most effectively with the topic distribution for providing the best classification performance.

The main aim of TextNetTopics Pro is to reduce the original text’s dimensionality and make the short text less sparse and more topic-oriented for classification purposes.

By using semantically richer document representation, TextNetTopics Pro can distinguish alternative forms expressing the same notion or concept. Therefore, it reduces the noise caused by synonymy and polysemy found in textual data.

**FIGURE 1:**
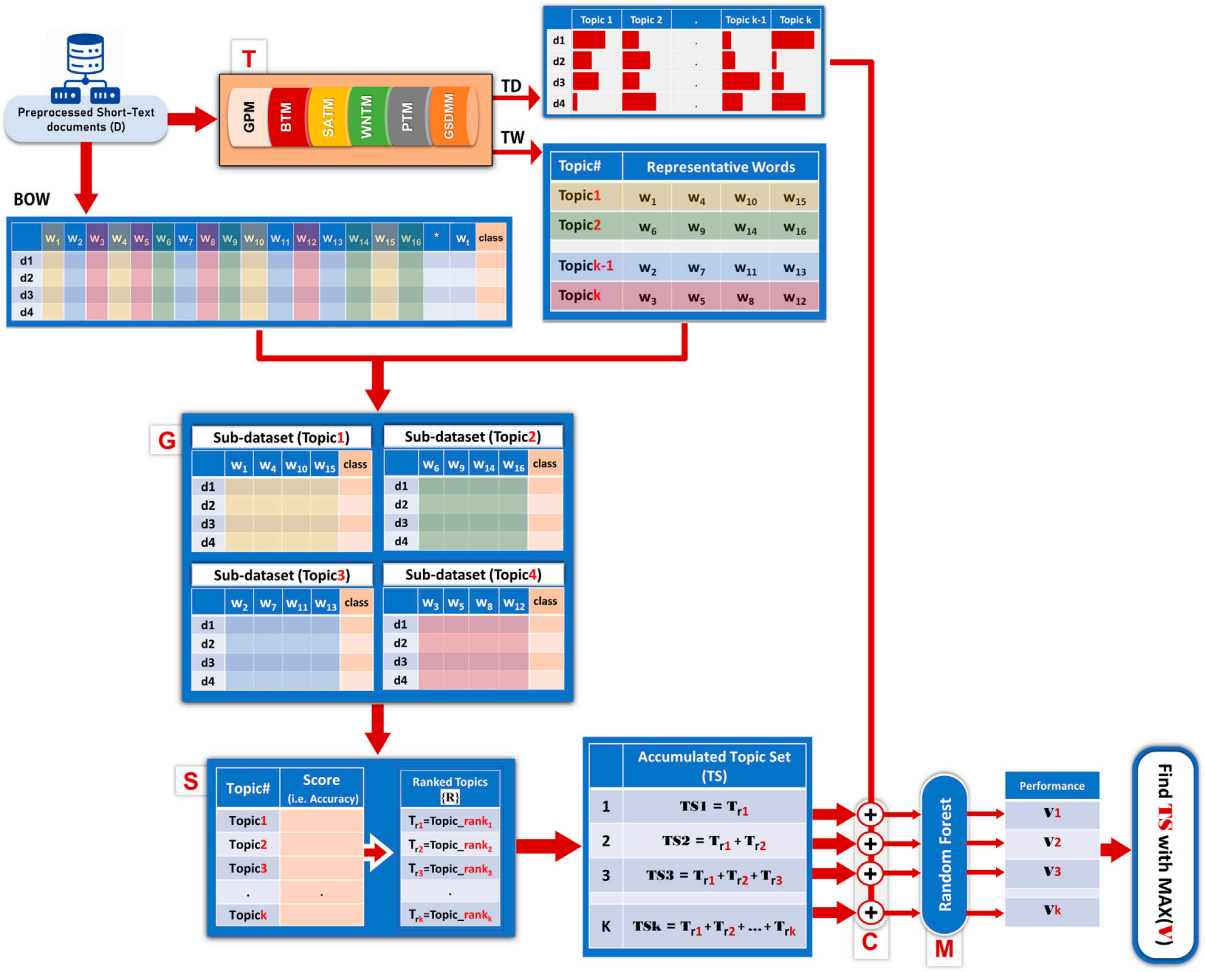
Workflow of TextNetTopics Pro.

Let D = {d_1_, d_2_, … , d_n_} be the collection of *n* short-text documents (See Figure 1, D collections).

Let TD be the matrix of document-topic distribution (See Figure 1, TD table), representing the likelihood or proportion of each topic present in a given document. The dimension of TD is *n* rows and *k* columns, where *k* is the number of detected topics.

Let TW be the topic_word matrix (See Figure 1, TW table), representing the distribution of words across topics, where each row corresponds to a topic and contains a set of *m* words that are semantically related within that topic.

TextNetTopics Pro algorithm consists of the following five main components, as shown in Figure 1.

- T component incorporates a short-text topic model (e.g., GSDM, BTM, *etc.*) to detect latent topics from the preprocessed collection of documents. The number of topics (k) and the number of terms per topic (m) are user-defined parameters. The main output of this component is two byproducts: a document-topic distribution matrix (TD) reflecting the topics’ proportions over documents and a topic-word distribution matrix (TW) where each word is assigned to each topic with probability.
- G component input is the topic_word matrix (TW) that represents the detected topics. For each topic that consists of m terms/words, the G component generates a representative two-class m-dimensional sub-dataset with its associated class labels from the training Bag-of-Words (BOW) table. In other words, each of these sub-datasets corresponds to a specific topic, containing only the terms that constitute that topic.
- S component utilizes an internal machine learning cross-validation applied to each representative two-class sub-dataset to assign a score or weight as the mean accuracy of the cross-validation. The score also might be another performance measurement, such as the area under the curve, and rank them accordingly. A *topic score* indicates the ability of words belonging to a topic to classify class labels.
- C component considers the top-ranked topics (topic = set of terms) in an accumulated fashion (referred to in Figure 1 as Topic Set (TS), e.g., top_1 ranked topic, top_1 + top_2 ranked topics, till top_1 to top_r ranked topics are merged, where r≤k), forming an aggregated subset of words, then extracts its two-class sub_dataset from the training BOW dataset and concatenates it with the TD matrix (same number of rows). This procedure is repeated cumulatively, creating r new datasets that we refer to each by C_TWD_i_, where i = 1 … r. Each C_TWD_i_ will serve as input to the M component.
- M component performs the training of the machine learning model (we use Random Forest) and the testing to create the performance table. From all the candidate subsets of features, we choose the optimal feature subset containing topics’ terms and distributions that provide the best performance (i.e., best discriminative power) with a reduced number of features for training the final classifier.

## 5 Experimental work

### 5.1 Datasets

In order to evaluate TextNetTopics and its enhanced version on short text documents, we conducted experiments on two publicly available datasets, the CAMDA dataset (CAMDA, 2022) and the arXiv dataset (arXiv Paper Abstracts, 2022).

1) *CAMDA dataset:* As part of a contest, the CAMDA panel has collected titles and abstracts of a large set of PubMed papers relevant to Drug-Induced Liver Injury (7,097 positive instances) and a challenging set of unrelated papers (7,026 negative instances). The DILI-related papers are validated by a committee of DILI specialists and referenced in the NIH LiverTox database. Additionally, in CAMDA the following datasets are provided:
  - Testing dataset (T1-T3): CAMDA has provided three unbalanced test sets at different difficulty levels. In other words, these datasets include an increasing amount of true negatives (where the majority of manuscripts are non-DILI related) to reflect the difficulty of the real-world task.
  - Validation (V1-V4): Four validation sets are provided at the end of the competition. In addition to hidden parts of T1-T3 datasets, CAMDA has prepared V4 - a domain transfer challenge to enable participants to assess the generalizability of the “DILI relevance detection” models.

CAMDA has provided Test Leaderboards that allow the user to upload a list of predictions of each test (T1-T3) and other Validation Leaderboards for validation datasets (V1-V4).

Table 1 provides information about the CAMDA training, testing, and validation datasets utilized in this study.

**TABLE 1:** Information on training, testing, and validation datasets used in this study.

	T_r_	T1	T2	T3	V1	V2	V3	V4
# records	∼1,400	4,763	21,724	82,753	6,494	32,814	100,265	1,400
a) Train		b) Test			c) Validation

2) *arXiv paper abstract dataset:* This dataset is found in Kaggle, and it is used for building multi-label text classifiers. In this study, we have converted this dataset into two balanced class dataset to evaluate our approach. We chose the Computer Vision and Pattern Recognition papers as a positive class (8,822 instances) and merged the remaining fields to create a negative class (8,341 instances). We performed stratified sampling for the non-relevant fields to retain their distribution in the final corpus.

Although both datasets consist of titles and abstracts of scientific publications, this study utilizes the titles’ part for experimental evaluation, except for the final section, which also uses the abstract part.

We plot the title length histogram to gain more insight into both datasets (refer to Supplementary Figures S1 and S2). The number of words in article titles tends to have a normal distribution. CAMDA titles have an average of 8.3 and a median of 8 words, while arXiv titles have an average of 6.9 and a median of 7 words. Supplementary Material includes detailed descriptive statistics for both datasets.

### 5.2 Preprocessing

This step is crucial to refine the text data to remove irrelevant, redundant, noninformative, and noisy data. Otherwise, their presence misleads the classifier, degrades performance, and substantially increases the computational time of machine learning. For the preprocessing task, we utilized the Knime workflows found in Yousef (2023) in order to perform the following Natural Language Processing operations: we removed all punctuations, numbers, non-alphanumeric characters, terms with less than three characters, and stop words (overly common terms which are neither descriptive nor meaningful and carry no semantic importance). We performed case-folding (lowercasing) and tokenization. Moreover, we stemmed text utilizing the Snowball Library, which aids vocabulary standardization. Finally, we used a minimal frequency cut-off to filter out the rare terms with a frequency of less than 20; these terms have no significant relevance and lack power in distinguishing different documents, yielding a total vocabulary of 1,293 terms for the CAMDA dataset and 1,175 terms for the arXiv dataset that are thematically unique and descriptive. However, we omitted the final step (minimum word-document frequency filter) for TextNetTopics Pro since we observed it degrades the final performance. Without this step, we can preserve more semantic information/structure in the text. After the preprocessing step, we utilized a term-weighting method called relative Term Frequency format, where each value in a document vector results from the division of the respective term count by the total number of terms in a document.

### 5.3 Experimental setup

To extract the topic-word matrix and the latent semantic representation (document-topic matrix) from the above-mentioned state-of-the-art algorithms (GSDMM, GPU-DMM, GPU-PDMM, LFDMM, BTM, PTM, SATM, and WNTM), we used STTM (Version = 1.8) (Qiang et al., 2022; Qiang, 2023), an open-source java library for Short Text Topic Modeling. Pre-trained 200-dimensional GloVe (Pennington et al., 2014) (Global Vectors) word embeddings were utilized by DMM-based algorithms (GPU-DMM, GPU-PDMM, LF-DMM). Regarding the Gamma Poisson mixture model (GPM), we used GPyM_TM (Version = 3.0.1) (jrmazarura, 2022), a Python package, to perform topic modeling.

For all algorithms, we use fixed hyperparameters of α = 0.1 and β = 0.01. We run Gibbs sampling inference for 1,000 iterations in all methods to guarantee convergence, and the final samples are utilized to estimate model parameters. We set the number of topics and the number of words per topic to twenty since it yielded the best performance among variants.

Concerning TextNetTopics and TextNetTopics Pro, we utilized their KNIME workflow implementations, which can be found in (Yousef, 2023).

Finally, we employed a stratified Monte Carlo Cross-Validation (MCCV) to evaluate the performance of our approach and measure its statistical significance, repeated ten times. Each time, we divide the dataset into two parts: ninety percent for training and ten percent for testing. Using MCCV enables every observation in the dataset to have an opportunity of appearing in the training and testing set. We utilized stratified splitting, keeping the proportions of instances in each class equal.

### 5.4 Evaluation

We adopt the standard performance measures as the evaluation criteria for our proposed short-text classification framework, such as accuracy, recall, specificity, precession, the area under the curve, and the F1-score. However, in the experimental discussion section, we focused on F1-score as the primary metric for evaluation.

## 6 Experimental results and discussion

### 6.1 Performance evaluation of TextNetTopics using various short-text topic models

Given that the utilized short-text topic models yielded approximately similar performance patterns across the two datasets, we report only the results obtained for the CAMDA dataset in this section. However, we included the results attained by the arXiv dataset as Supplementary Material.

Table 2 reports the performance of TextNetTopics for the CAMDA dataset when incorporating various short-text topic models in the T component. In this table, the highlighted cells represent the maximum F1-score achieved by each topic model. According to the F1-score results, we see robustness in feature subset generation by TextNetTopics. The change in the performance linearly increases as we increase the feature subset generated by TextNetTopics. This behavior confirms our feature selection method’s stability and ability to identify the most relevant and discriminative topical word features at any subset that optimizes short-text classification. In other words, TextNetTopics is able to retain only terms that improve, or at least do not hinder, prediction performance.

**TABLE 2:** TextNetTopics performance over accumulated top-ranked topics in the CAMDA dataset, utilizing the various short-text topic modeling methods in the T component.

	# of Accumlated_Topics	# of terms (mean)	Accuracy (mean)	Recall (mean)	Specificity (mean)	F-measure (mean)	AUC (mean)	Precision (mean)	Cohen’s kappa (mean)
**GSDMM**	20	185	85.10	84.75	85.46	85.14	90.34	85.54	70.21
	18	167	85.15	84.17	86.13	85.08	90.16	86.03	70.29
	16	149	85.12	83.52	86.75	84.97	90.21	86.48	70.25
	14	133.3	84.69	82.70	86.70	84.47	89.95	86.33	69.39
	12	117.8	84.49	82.26	86.75	84.22	89.63	86.30	68.99
	10	103	83.71	81.19	86.26	83.38	88.82	85.70	67.43
	8	84	83.04	80.37	85.74	82.67	88.52	85.12	66.09
	6	67	82.80	79.62	86.02	82.32	87.89	85.24	65.61
	4	48.4	81.18	76.70	85.72	80.40	86.00	84.49	62.38
	3	40	80.64	75.39	85.96	79.67	85.14	84.50	61.31
	2	32.4	79.58	73.68	85.56	78.40	83.56	83.82	59.19
	1	20	76.44	67.48	85.52	74.23	79.91	82.58	52.94
**PTM**	20	275	85.95	86.31	85.59	86.07	91.99	85.85	71.90
	18	244	86.04	85.73	86.36	86.07	91.87	86.44	72.09
	16	219.6	85.86	85.30	86.43	85.86	91.66	86.44	71.73
	14	193	85.59	84.50	86.69	85.51	91.30	86.55	71.18
	12	171	85.35	83.68	87.03	85.18	90.86	86.75	70.70
	10	145.3	84.83	82.67	87.02	84.58	90.32	86.59	69.67
	8	120.5	83.80	81.24	86.39	83.46	89.33	85.83	67.61
	6	93	81.94	77.85	86.07	81.26	87.02	85.00	63.89
	4	67.2	77.69	71.20	84.27	76.26	81.95	82.12	55.42
	3	54	76.66	69.58	83.84	75.00	80.77	81.36	53.37
	2	37.7	74.33	64.37	84.43	71.60	77.59	80.73	48.73
	1	20	69.74	55.47	84.20	64.84	72.33	78.11	39.60
**BTM**	20	194	85.45	84.78	86.13	85.43	90.71	86.10	70.91
	18	182	85.61	84.65	86.58	85.55	90.63	86.47	71.22
	16	170.5	85.41	83.97	86.86	85.27	90.62	86.62	70.82
	14	149.8	85.01	82.90	87.15	84.77	90.04	86.73	70.03
	12	131	84.85	82.60	87.13	84.58	89.95	86.67	69.72
	10	114.6	84.53	82.15	86.93	84.23	89.65	86.43	69.06
	8	93.5	83.92	81.03	86.85	83.53	89.09	86.19	67.85
	6	71.5	83.37	80.34	86.45	82.94	88.25	85.73	66.76
	4	53.6	82.30	78.59	86.06	81.71	86.76	85.10	64.61
	3	42.5	79.94	73.71	86.26	78.70	83.54	84.45	59.92
	2	32	76.71	66.68	86.88	74.23	79.84	83.74	53.48
	1	20	75.81	66.80	84.93	73.54	78.59	81.84	51.67
**SATM**	20	245	85.72	86.12	85.30	85.85	91.68	85.59	71.43
	18	221.2	85.47	85.37	85.57	85.54	91.17	85.71	70.95
	16	200	85.46	84.55	86.38	85.41	90.85	86.29	70.92
	14	177	85.11	83.80	86.43	84.99	90.55	86.23	70.22
	12	158.3	84.88	83.30	86.49	84.72	89.96	86.22	69.77
	10	135.1	82.53	79.93	85.17	82.14	87.39	84.51	65.08
	8	111.4	81.17	77.19	85.20	80.47	85.92	84.06	62.35
	6	90	79.67	75.15	84.26	78.81	83.85	82.87	59.37
	4	64	77.99	71.73	84.33	76.62	81.96	82.27	56.01
	3	50.8	76.78	69.32	84.34	75.01	80.63	81.78	53.61
	2	36	75.48	66.14	84.94	73.07	78.87	81.66	51.02
	1	20	73.74	62.38	85.24	70.49	76.84	81.10	47.55
**WNTM**	20	220	84.27	84.13	84.41	84.33	89.89	84.55	68.54
	18	199	84.22	83.58	84.87	84.20	89.74	84.86	68.44
	16	181	84.26	83.28	85.26	84.19	89.45	85.15	68.53
	14	161	84.00	82.21	85.82	83.79	89.43	85.48	68.01
	12	142.1	83.47	81.58	85.39	83.25	89.14	84.99	66.95
	10	119.9	83.39	81.26	85.54	83.12	88.77	85.08	66.78
	8	99.8	82.58	79.97	85.21	82.20	87.85	84.60	65.16
	6	80.3	82.04	78.59	85.54	81.50	86.83	84.67	64.10
	4	57.2	80.75	76.25	85.30	79.94	85.52	84.04	61.52
	3	46	79.27	73.58	85.03	78.12	84.07	83.29	58.56
	2	32.3	76.31	69.49	83.21	74.69	80.46	80.76	52.65
	1	20	75.84	67.68	84.10	73.81	79.53	81.20	51.72
**GPM**	18	200	85.00	84.33	85.67	84.98	90.54	85.68	69.99
	16	164	84.70	83.82	85.60	84.65	90.16	85.54	69.41
	14	145	84.75	83.37	86.16	84.62	90.18	85.95	69.51
	12	128	84.55	82.87	86.25	84.37	90.00	85.95	69.10
	10	112.4	84.46	82.36	86.58	84.21	89.64	86.16	68.92
	8	97.4	83.88	81.56	86.23	83.58	89.16	85.73	67.77
	6	77	83.02	79.75	86.35	82.54	87.61	85.57	66.06
	4	54.7	82.11	77.43	86.85	81.32	86.70	85.66	64.23
	3	45.1	81.39	76.05	86.79	80.44	85.89	85.38	62.80
	2	34	79.79	73.61	86.05	78.56	84.02	84.25	59.60
	1	20	76.39	64.91	88.02	73.45	79.53	84.60	52.85
**GPU_DMM**	20	166	85.09	84.96	85.21	85.15	90.40	84.96	85.36
	18	150.5	84.96	84.31	85.62	84.94	90.30	84.31	85.61
	16	136.7	84.67	83.56	85.79	84.57	90.21	83.56	85.64
	14	122.4	84.38	82.62	86.16	84.18	89.83	82.62	85.84
	12	110	84.05	82.39	85.73	83.86	89.25	82.39	85.42
	10	90.6	83.61	81.65	85.59	83.37	89.10	81.65	85.18
	8	78.2	83.35	81.36	85.37	83.10	88.49	81.36	84.94
	6	63.5	82.61	79.90	85.36	82.22	87.69	79.90	84.69
	4	46	80.77	75.86	85.74	79.87	85.58	75.86	84.37
	3	40	80.11	74.34	85.96	78.99	84.51	74.34	84.30
	2	32.6	79.30	72.87	85.82	77.97	83.37	72.87	83.88
	1	19.9	76.63	67.17	86.20	74.30	80.17	67.17	83.18
**GPU_PDMM**	20	276	85.02	85.88	84.15	85.23	91.06	84.59	70.05
	18	249	85.03	85.36	84.70	85.16	91.07	84.97	70.06
	16	223	85.07	84.98	85.16	85.14	90.86	85.30	70.13
	14	194.6	85.08	84.57	85.60	85.09	90.62	85.62	70.16
	12	173.6	84.80	83.55	86.07	84.69	90.52	85.88	69.61
	10	150	84.55	82.91	86.20	84.37	90.11	85.89	69.10
	8	122.5	83.30	80.42	86.20	82.88	88.38	85.53	66.60
	6	93.8	82.33	78.50	86.22	81.72	87.07	85.24	64.69
	4	63.1	77.75	70.78	84.81	76.18	82.01	82.52	55.54
	3	48	77.15	69.73	84.67	75.43	80.98	82.19	54.35
	2	35	76.36	67.99	84.84	74.32	79.74	81.98	52.78
	1	20	70.14	53.44	87.05	64.26	72.08	81.07	40.40
**LFDMM**	20	202	85.22	84.91	85.54	85.25	90.50	85.62	70.45
	18	180.6	85.27	84.46	86.09	85.22	90.49	86.02	70.54
	16	164.8	85.08	84.02	86.16	85.00	90.24	86.02	70.17
	14	148	84.93	83.34	86.55	84.77	89.99	86.26	69.87
	12	126.9	84.66	82.46	86.89	84.40	89.77	86.44	69.33
	10	112.1	84.12	81.56	86.72	83.78	89.43	86.16	68.25
	8	92.5	83.57	80.81	86.36	83.18	88.64	85.73	67.14
	6	71	82.71	79.34	86.13	82.20	87.49	85.29	65.44
	4	53.8	81.36	76.32	86.46	80.47	85.94	85.11	62.74
	3	44.3	80.53	74.31	86.82	79.32	84.85	85.09	61.08
	2	32	77.35	68.70	86.10	75.31	81.31	83.35	54.74
	1	19.5	74.90	64.17	85.76	71.96	78.31	82.08	49.86

**FIGURE 2:**
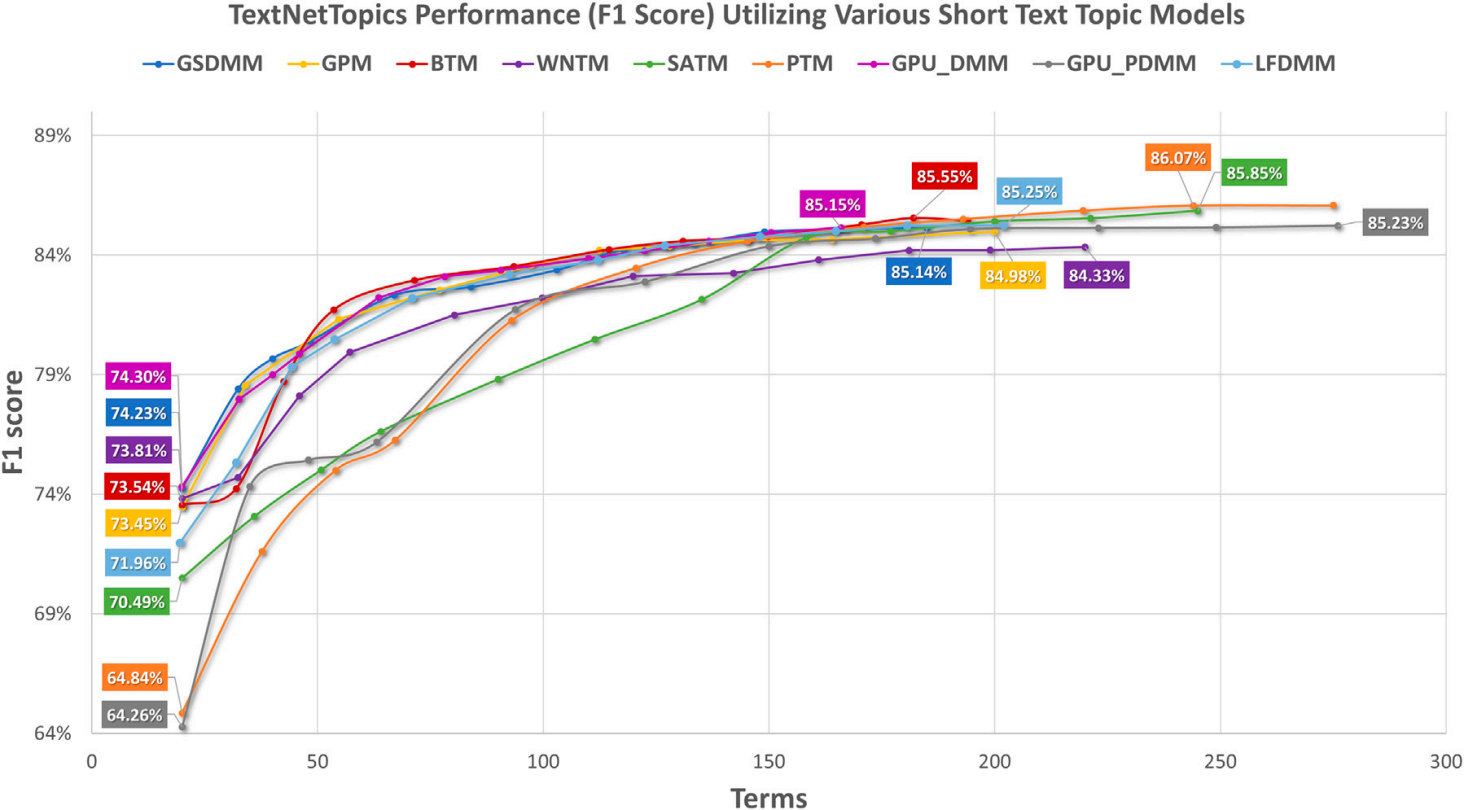
TextNetTopics performance over accumulated top-ranked topics for the CAMDA dataset using various short-text topic models in the T component. Symbols along the line represent the number of accumulated topics.

**FIGURE 3:**
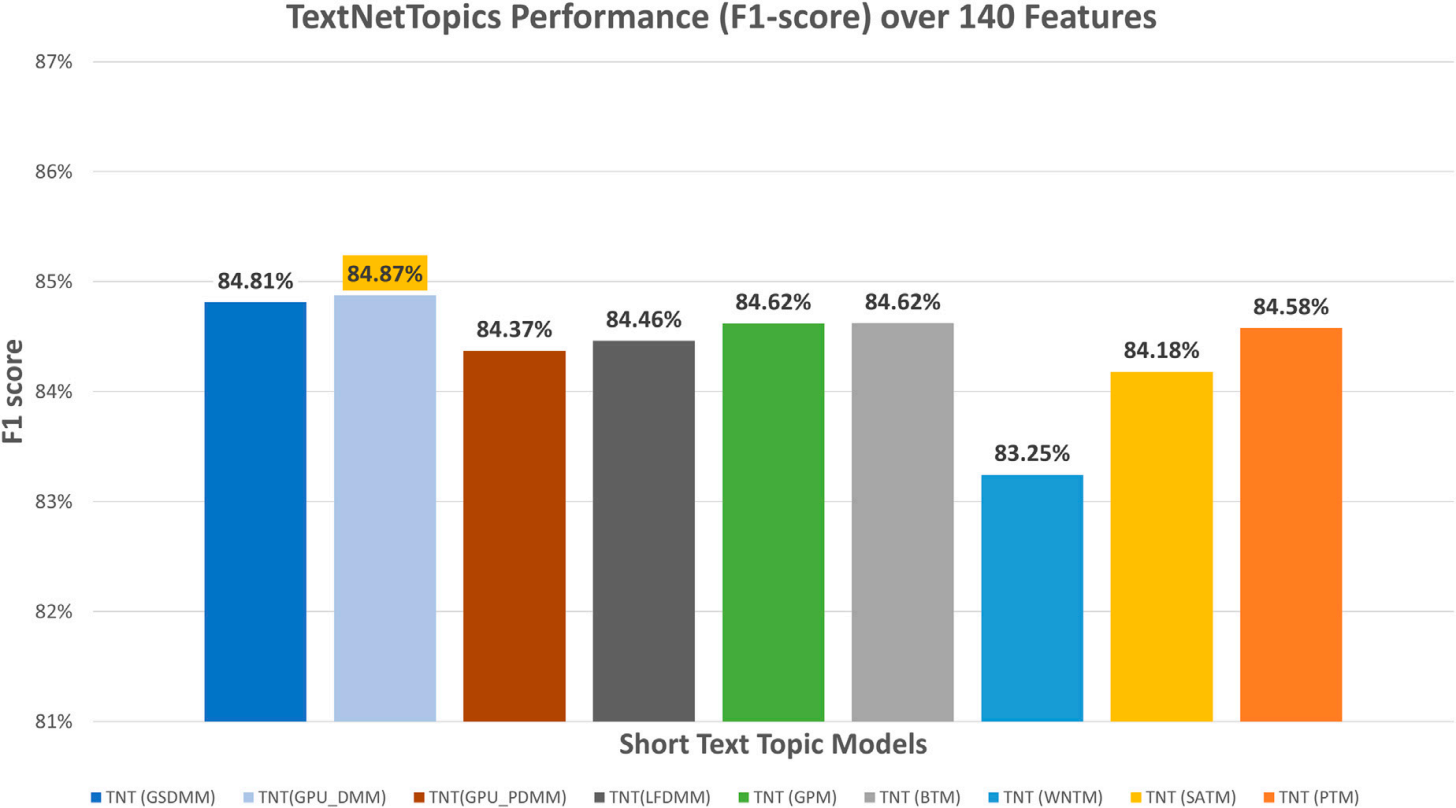
TextNetTopics performance over 140 features/terms for the CAMDA dataset using various short-text topic models in the T component.

Figure 2 presents the performance results of TextNetTopics over accumulated top topics (topical word subsets) for the DILI-CAMDA dataset using various short-text topic modeling methods in the T component. According to Figure 2, Self-aggregation methods achieved the highest F1 score. For instance, PTM got 86.07% over the top 19 topics with 257 terms, and SATM got 85.85% over the top 20 topics with 245 terms. Then BTM got 85.55% over the top 18 topics with 182 terms. DMM-based models achieved 85.25%, 85.23%, 85.15%, and 85.13% for LFDMM, GPU-PDMM, GPU-DMM, and GSDMM over the top 20 topics with 202, 276, 166, and 185 terms, respectively. Regarding the remaining topic models, although they resulted in comparable results, their F1 score was the lowest. For example, GPM and WNTM got 84.98% and 84.33% over the top 18 topics with 200 terms and 20 topics with 220 terms, respectively. According to Figure 3, when considering only 140 features, the GPU-DMM topic modeling method reported the highest performance F1-score result (84.87%) in the CAMDA dataset.

**FIGURE 4:**
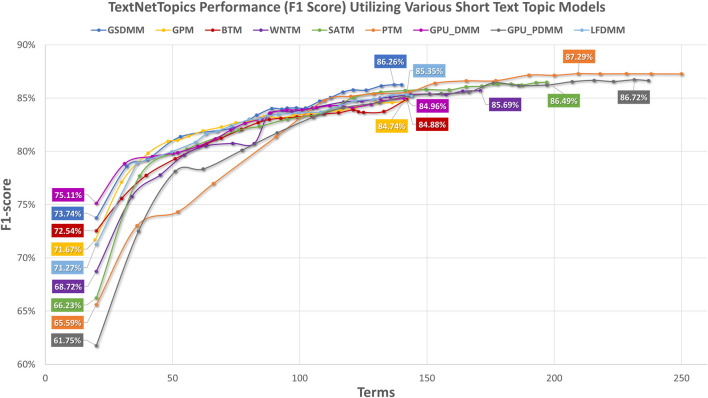
TextNetTopics performance over accumulated top-ranked topics for the arXiv dataset using various short-text topic models in the T component. Symbols along the line represent the number of accumulated topics.

**FIGURE 5:**
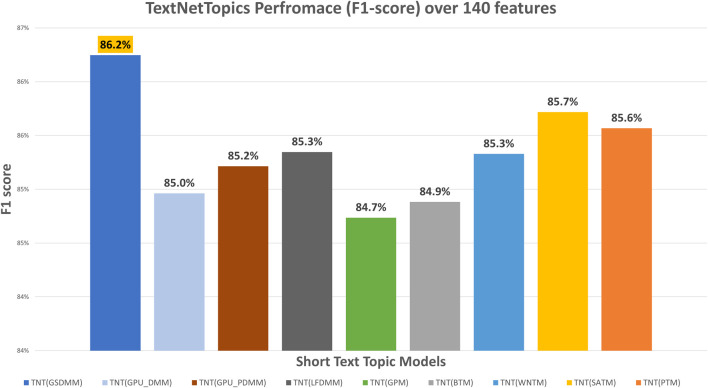
TextNetTopics performance over 140 features/terms for the arXiv dataset using various short-text topic models in the T component.

Figure 4 presents the performance results of TextNetTopics over accumulated top topics (topical word subsets) for the arXiv dataset using various short-text topic modeling methods in the T component. According to Figure 4, again, the Self-aggregation method and GPU-PDMM achieved the highest F1 score. For instance, PTM got 87.29% over the top 15 topics with 209 terms, GPU-PDMM and SATM achieved 86.64% and 86.49% over the top 20 topics with 237 and 197 terms. Then GSDMM got 86.26% over the top 19 topics with 137 terms, WNTM achieved 85.69% over the top 20 topics with 171 terms, and LFDMM got 85.35% over the top 19 topics with 142 terms. Regarding the remaining topic models, although they resulted in comparable results, their F1 score was the lowest. For example, GPU-DMM, BTM, and GPM got 84.96%, 84.88%, and 84.74% over the top 20 topics, with 141, 142, and 141 terms, respectively. According to Figure 5, when considering only 140 features, the highest F1-score was reported by the GSDMM topic modeling method (86.2%) in the arXiv dataset.

To this end, we attribute the minor differences in the performance of all the short-text topic models to the number of overlapped terms. Tables 3, 4 provide the number of shared terms over twenty topics between the topics extracted from the mentioned models. Interestingly, GPM in both datasets got the lowest intersected terms with others.

**TABLE 3:** The percentage of shared terms over twenty extracted topics between various short-text topic modeling methods in the CAMDA dataset. The diagonal values highlighted in bold represent the number of unique terms in twenty topics extracted by each short-text topic modeling method.

	GSDMM	GPM	BTM	WNTM	SATM	PTM	GPU_DMM	GPU_PDMM	LFDMM
**GSDMM**	**185**	49%	80%	81%	85%	92%	78%	93%	78%
**GPM**	44%	**205**	44%	43%	56%	56%	46%	55%	49%
**BTM**	76%	46%	**194**	85%	84%	90%	71%	89%	75%
**WNTM**	67%	40%	75%	**221**	74%	81%	65%	81%	64%
**SATM**	64%	47%	66%	66%	**246**	81%	60%	80%	63%
**PTM**	62%	41%	63%	65%	72%	**276**	57%	80%	62%
**GPU_DMM**	82%	54%	78%	81%	84%	90%	**176**	94%	79%
**GPU_PDMM**	62%	41%	63%	64%	72%	80%	60%	**276**	61%
**LFDMM**	69%	47%	69%	67%	74%	81%	66%	80%	**211**

**TABLE 4:** The percentage of shared terms over twenty extracted topics between various short-text topic modeling methods in the arXiv dataset. The diagonal values highlighted in bold represent the number of unique terms in twenty topics extracted by each short-text topic modeling method.

	GSDMM	GPM	BTM	WNTM	SATM	PTM	GPU_DMM	GPU_PDMM	LFDMM
**GSDMM**	**140**	80%	83%	90%	91%	98%	90%	97%	81%
**GPM**	75%	**149**	72%	78%	79%	87%	77%	87%	72%
**BTM**	82%	75%	**142**	91%	92%	98%	80%	96%	77%
**WNTM**	73%	67%	75%	**172**	83%	92%	73%	92%	70%
**SATM**	65%	60%	66%	72%	**197**	91%	64%	91%	63%
**PTM**	55%	52%	56%	63%	72%	**250**	55%	86%	52%
**GPU_DMM**	89%	81%	80%	89%	90%	97%	**141**	98%	80%
**GPU_PDMM**	57%	55%	58%	67%	75%	90%	58%	**238**	55%
**LFDMM**	75%	70%	72%	79%	82%	86%	74%	87%	**152**

### 6.2 Comparative performance evaluation of TextNetTopics with other feature selection algorithms

We have comparatively evaluated TextNetTopic utilizing various short-text topic models with three different feature selection methods, namely, selectKBest (SKB), Fast Correlation Based Filter (FCBF), and Extreme Gradient Boosting (XGBoost), using four different classifiers: Adaboost, Decision Tree, Random Forest, and LogitBoost. We present the results in Tables 5, 6, where the highest scores are highlighted in bold for each metric. We have included the topic models that have generated a significant number of unique words, ensuring that each model produced more than 180 features. This selection criterion was implemented to ensure that the topic models provided an adequate amount of distinct information for analysis and classification purposes.

**TABLE 5:** Comparative performance evaluation of TextNetTopic with various feature selection algorithms using 180 features in the CAMDA dataset.

Feature selection	Topic model	ML model	Accuracy	Recall	Specificity	F1	AUC	Precision
**TextNetTopic**	GSDMM	RF	85.10	84.75	85.46	85.14	90.34	85.54
	PTM		**85.62**	84.34	**86.92**	85.51	91.30	**86.74**
	BTM		85.61	84.65	86.58	85.55	90.63	86.47
	SATM		85.28	84.54	86.03	85.25	90.65	85.99
	WNTM		84.26	83.28	85.26	84.19	89.45	85.15
	GPM		84.80	84.38	85.23	84.82	90.21	85.31
	GPU_PDMM		85.12	84.44	85.80	85.10	90.59	85.77
	LFDMM		85.27	84.46	86.09	85.22	90.49	86.02
**XGBOOST**		Adaboost	84.43	84.64	84.23	84.56	91.44	84.69
		DT	83.10	81.50	84.73	82.92	85.42	84.43
		LogitBoost	84.67	83.39	85.96	84.56	91.08	85.82
		RF	85.40	86.28	84.50	**85.60**	**91.58**	84.96
**SKB**		Adaboost	84.56	85.88	83.21	83.92	91.69	84.85
		DT	82.06	81.49	82.65	82.68	84.86	82.06
		LogitBoost	84.83	85.80	83.84	84.38	91.43	85.06
		RF	84.93	86.66	83.17	83.95	91.73	85.27
**FCBF**		Adaboost	53.55	96.36	10.19	67.64	48.04	52.15
		DT	50.32	**100.00**	0.00	66.95	46.07	50.32
		LogitBoost	53.57	97.30	9.27	67.86	47.23	52.14
		RF	50.81	99.34	1.66	67.03	46.08	50.60

**TABLE 6:** Comparative performance evaluation of TextNetTopic with various feature selection algorithms using 180 features in the arXiv dataset.

Feature selection	Topic model	ML model	Accuracy	Recall	Specificity	F1	AUC	Precision
**TextNetTopic**	PTM		87.80	85.21	90.25	87.16	93.07	89.22
	SATM		87.14	83.55	90.53	86.33	92.36	89.32
	WNTM		86.42	83.65	89.05	85.69	92.26	87.85
		Adaboost	87.79	83.49	91.86	86.92	94.04	90.69
		DT	86.44	83.15	89.56	85.62	89.79	88.33
		LogitBoost	87.80	83.91	91.48	86.99	93.82	90.33
		RF	**87.97**	85.04	90.74	**87.29**	93.91	89.71
SKB		Adaboost	87.79	83.43	**92.10**	87.00	**94.38**	**90.93**
		DT	85.75	83.27	88.09	85.03	88.91	86.89
		LogitBoost	87.78	83.66	91.68	86.94	94.21	90.50
		RF	87.75	84.74	90.79	87.14	93.98	89.70
FCBF		Adaboost	55.00	94.73	17.43	67.33	58.20	52.56
		DT	48.60	**100.00**	0.00	65.41	55.68	48.60
		LogitBoost	54.68	94.79	16.75	67.15	56.24	52.29
		RF	52.60	94.48	13.00	66.01	55.92	50.95

In the CAMDA dataset, TextNetTopics with the PTM topic model achieves the highest accuracy, specificity, and precision. Although XGBOOST with Random Forest got the highest F1-score and AUC, TextNetTopics has comparable results. FCBF got the lowest performance scores (except recall), which reflects its inability to handle short text.

In the arXiv dataset, XGBOOST with Random Forest and TextNetTopics with the PTM topic model achieves comparable accuracy, recall, and F1-score performance. Regarding specificity, AUC, and precision, SKB got the highest results. Concerning FCBF, it got the lowest performance scores (except recall), reflecting its inability to handle short text.

The results obtained for TextNetTopics are reasonable since each short text contains a limited number of words after preprocessing, e.g., the omission of stop words, which leads to the scarcity of discriminative features. In addition, the available information, such as word frequency, is insufficient for classification, and these problems inevitably compromise the quality of a classifier. Thus, using only BOW representation with top-weighted topics is insufficient to boost the short-text classification performance.

### 6.3 Performance evaluation of TextNetTopics Pro

In contrast to TextNetTopics, TextNetTopics Pro performs semantic extension, combining top topics’ words with the topic distribution generated by the topic model. Such features captured at the corpus level preserve the relationship information of words with similar meanings and alleviate data sparsity problems which in turn improves the classification efficiency.

Tables 7, 8 report the classification performance in the CAMDA dataset and arXiv dataset when utilizing topical words extracted by TextNetTopics, topic distribution features generated by Topic Models, all terms (BOW) combined with topic distribution features, and our proposed approach, which combines words of top-ranked topics extracted by TextNetTopics with topic distribution features. In these tables, the highest scores are highlighted in bold for each metric.

**TABLE 7:** Performance results of various document representations (CAMDA dataset). TD refers to topic distributions extracted by a topic model, TW refers to topical words subset (accumulated top_ranked topics) selected by TextNetTopics, and BW refers to Bag-of-Words or all the terms in the preprocessed dataset. TW + TD refers to our proposed approach.

Topic model	Features type	# of features	Accuracy	Sensitivity	Specificity	Precision	F1-measure	Cohen’s kappa
GSDMM	TW + TD	193	89.65	90.99	88.29	88.79	89.87	79.30
	BW + TD	1,312	89.65	91.02	88.27	88.72	89.85	79.30
	TW	185	85.10	84.75	85.46	85.54	85.14	70.21
	TD	20	87.87	88.98	86.74	87.24	88.09	75.73%
BTM	TW + TD	167	90.04	90.25	89.83	90.04	90.14	80.08
	BW + TD	1,312	90.23	89.89	**90.59**	**90.64**	90.26	80.47
	TW	182	85.61	84.65	86.58	86.47	85.55	71.22
	TD	20	89.05	89.38	88.71	88.93	89.14	78.09
PTM	TW + TD	254	89.38	89.71	89.05	89.29	89.49	78.75
	BW + TD	1,312	89.67	89.52	89.83	89.92	89.72	79.34
	TW	257	86.04	85.87	86.22	86.33	86.09	72.09
	TD	20	87.32	86.46	88.18	88.12	87.28	74.64
SATM	TW + TD	245	85.73	84.48	86.99	86.86	85.75	71.46
	BW + TD	1,312	86.56	85.50	87.64	87.52	86.49	73.13
	TW	245	85.72	86.12	85.30	85.59	85.85	71.43
	TD	20	80.93	80.06	81.81	80.06	80.86	61.85
WNTM	TW + TD	155	85.78	85.49	86.07	86.19	85.83	71.55
	BW + TD	1,312	87.04	87.10	86.98	87.15	87.12	74.08
	TW	220	84.27	84.13	84.41	84.55	84.33	68.54
	TD	20	85.10	85.39	84.80	85.12	85.24	70.19
GPM	TW + TD	191	88.67	88.68	88.66	88.84	88.75	
	BW + TD	1,312	89.74	89.38	90.10	90.15	89.76	79.47
	TW	220	85.00	84.33	85.67	85.68	84.98	69.99
	TD	20	84.44	81.37	87.55	86.89	84.03	68.89
GPU_DMM	TW + TD	160.7	90.11	91.67	88.52	89.05	90.33	80.21
	BW + TD	1,312	**90.38**	**92.52**	88.22	88.84	**90.64**	**80.76**
	TW	166	85.02	85.88	84.15	84.59	85.23	70.05
	TD	20	88.30	89.15	87.44	87.84	88.48	76.59
GPU_PDMM	TW + TD	281	89.36	89.26	89.47	89.63	89.43	78.73
	BW + TD	1,312	89.06	88.36	89.77	89.75	89.04	78.12
	TW	276	85.02	85.88	84.15	84.59	85.23	70.05
	TD	20	88.13	88.17	88.09	88.29	88.22	76.26
LFDMM	TW + TD	201	89.03	89.03	89.02	89.19	89.11	78.05
	BW + TD	1,312	89.22	89.46	88.97	89.15	89.30	78.43
	TW	191	85.37	84.84	85.92	85.93	85.37	70.75
	TD	20	87.97	88.32	87.62	87.90	88.10	75.94

**TABLE 8:** Performance results of various document representations (on arXiv dataset). TD refers to topic distributions extracted by a topic model, TW refers to topical words subset (accumulated top_ranked topics) selected by TextNetTopics, and BW refers to Bag-of-Words or all the terms in the preprocessed dataset. TW + TD refers to our proposed approach.

Topic model	Features type	# of features	Accuracy	Sensitivity	Specificity	Precision	F1-measure	Cohen’s kappa
GSDMM	TW + TD	152	**90.43**	88.71	92.06	91.35	**90.01**	**80.83**
	BW + TD	1,195	90.34	88.06	92.50	91.74	89.86	80.64
	TW	140	87.11	83.19	90.82	89.56	86.25	74.15
	TD	20	87.80	86.00	89.51	88.58	87.26	75.57
BTM	TW + TD	142	90.09	**89.04**	91.09	90.42	89.72	80.16
	BW + TD	1,195	89.72	88.45	90.92	90.21	89.32	79.41
	TW	142	85.77	82.21	89.14	87.77	84.88	71.47
	TD	20	88.96	88.32	89.56	88.89	88.60	77.89
PTM	TW + TD	242.7	88.61	88.51	88.71	88.12	88.31	77.21
	BW + TD	1,195	88.97	87.73	90.15	89.39	88.55	77.92
	TW	209.4	87.86	85.82	89.78	88.83	87.29	75.68
	TD	20	85.52	85.55	85.49	84.80	85.16	71.03
SATM	TW + TD	182	86.07	82.51	89.43	88.07	85.19	72.07
	BW + TD	1,195	87.26	83.96	90.39	89.20	86.50	74.47
	TW	197	87.22	84.19	90.09	88.94	86.49	74.39
	TD	20	79.98	77.01	82.77	80.86	78.89	59.87
WNTM	TW + TD	181.5	87.16	83.82	90.31	89.10	86.38	74.25
	BW + TD	1,195	87.51	83.83	90.99	89.80	86.70	74.95
	TW	171	86.42	83.65	89.05	87.85	85.69	72.79
	TD	20	86.00	82.55	89.26	87.91	85.14	71.94
GPM	TW + TD	129	89.10	87.53	90.59	89.78	88.64	78.17
	BW + TD	1,195	90.29	88.07	92.40	91.63	89.81	80.55
	TW	141	85.67	81.88	89.24	87.80	84.74	71.25
	TD	20	86.86	85.64	88.02	87.11	86.36	73.69
GPU_DMM	TW + TD	117.5	90.39	87.95	92.70	**91.92**	89.89	80.74
	BW + TD	1,195	90.22	87.60	**92.70**	91.91	89.70	80.40
	TW	141	85.87	82.15	89.39	87.98	84.96	71.67
	TD	20	89.64	86.82	92.30	91.42	89.06	79.23
GPU_PDMM	TW + TD	230	88.91	87.06	90.65	89.79	88.40	77.77
	BW + TD	1,195	89.33	87.77	90.81	90.04	88.88	78.63
	TW	142	86.22	82.57	89.67	88.33	85.35	72.37
	TD	20	87.19	85.97	88.34	87.44	86.70	74.34
LFDMM	TW + TD	96	88.70	86.27	91.00	90.06	88.12	77.36
	BW + TD	1,195	88.74	86.18	91.16	90.22	88.15	77.43
	TW	142	86.22	82.57	89.67	88.33	85.35	72.37
	TD	20	87.49	85.25	89.60	88.57	86.88	74.93

**FIGURE 6:**
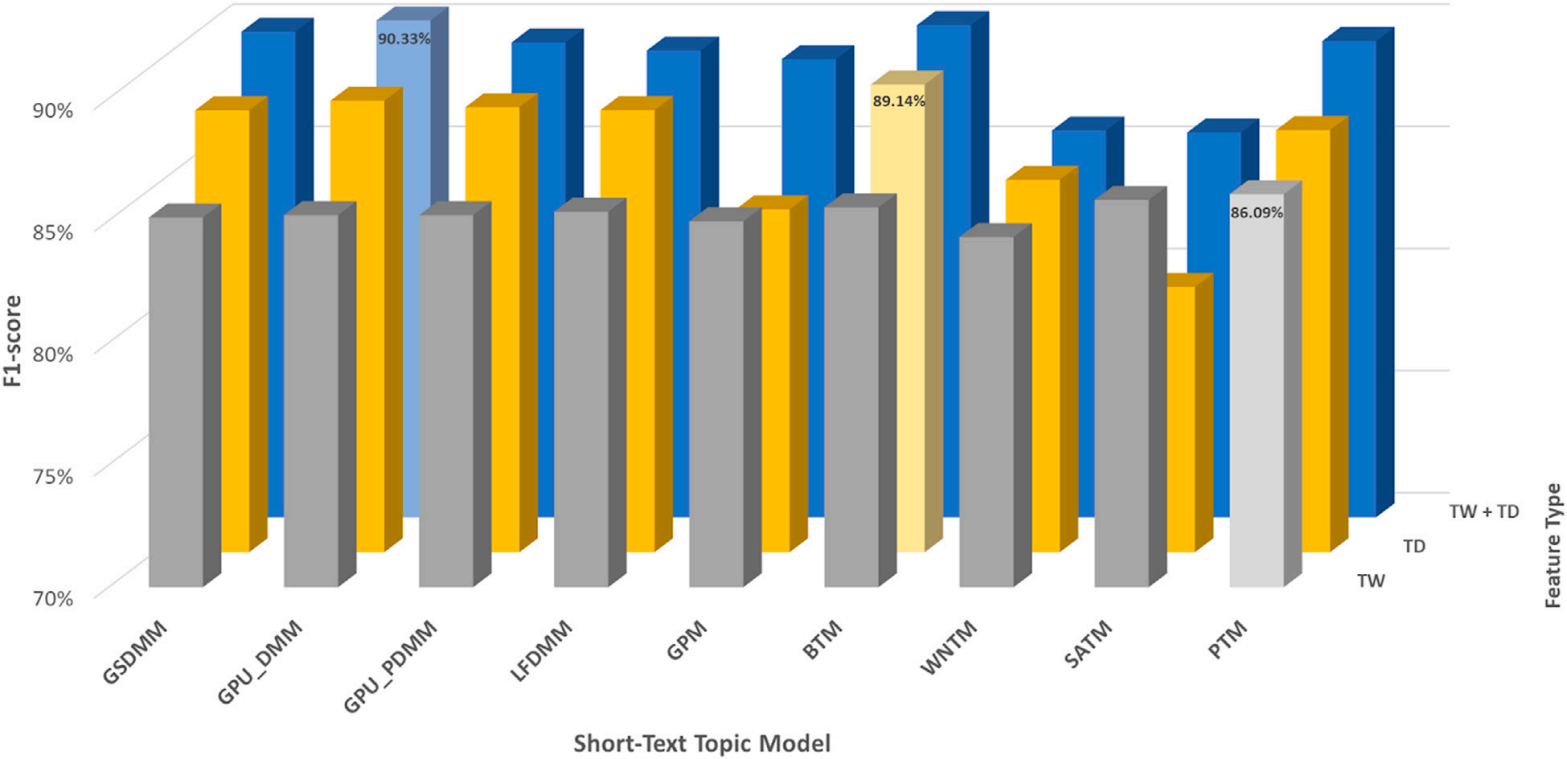
Classification performance of CAMDA dataset when utilizing topical words (TW) extracted by TextNetTopics, topic distribution features (TD) generated by Topic Models, and our proposed approach, combining words of top-ranked topics extracted by TextNetTopics with topic distribution features (TW + TD). The light-colored columns represent the highest achieved values.

In the CAMDA dataset, according to Table 7 and Figure 6, TextNetTopics Pro enhances the F1-score performance of TextNetTopics by 5.10%, 4.73%, 4.59%, 4.20%, 3.78%, 3.73%, 3.40%, and 1.50% when utilizing GPU_DMM, GSDMM, BTM, GPU_PDMM, GPM, LFDMM, PTM, and WNTM topic models, respectively. For SATM, we got similar results to TextNetTopics with no significant improvement. In addition, our proposed approach enhances the F1-score performance gained by topic distribution generated with SATM, GPM, PTM, GPU_DMM, GSDMM, GPU_PDMM, LFDMM, BTM, and WNTM topic models by 4.89%, 4.72%, 2.21%, 1.85%, 1.78%, 1.22%, 1.01%, 1.00%, and 0.59%, respectively.

**FIGURE 7:**
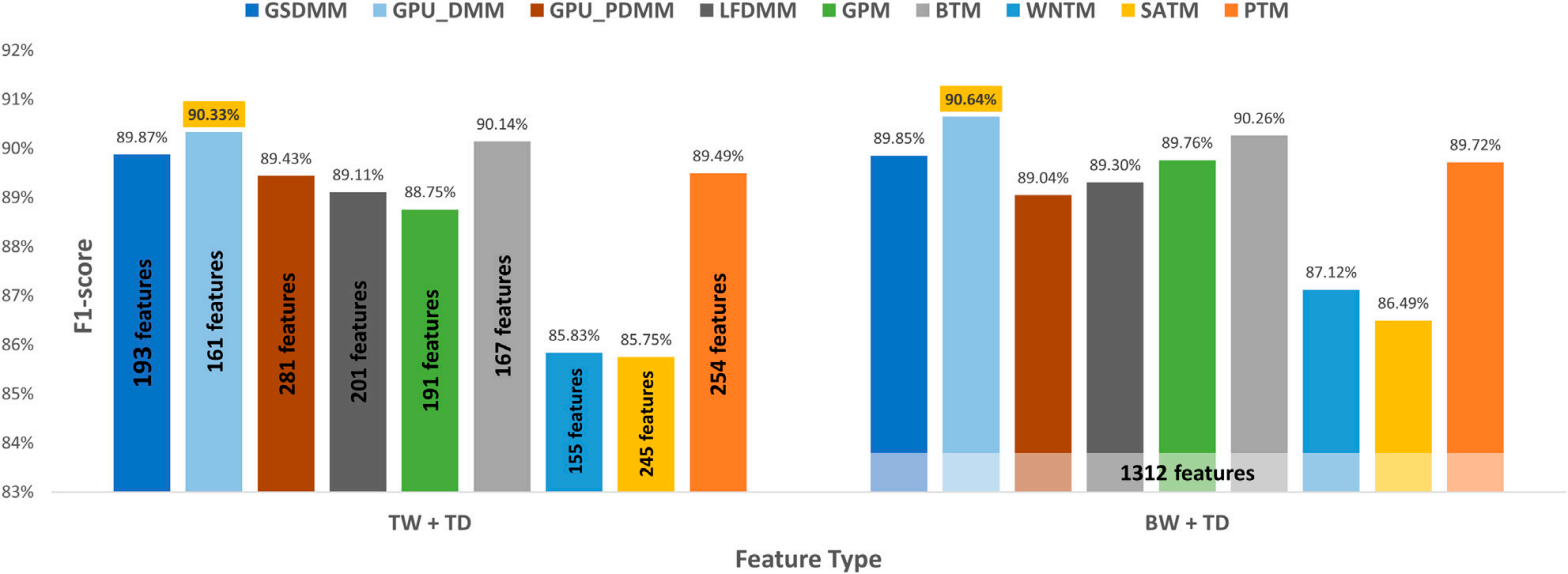
Classification performance of our proposed approach over the CAMDA dataset, compared with taking all preprocessed terms with the semantic features.

Moreover, we compared the performance results obtained when taking all terms in the preprocessed dataset combined with the semantic features (topic distribution extracted by the topic model) with our enhanced tool (refer to Figure 7). TextNetTopics Pro got similar or a slight improvement in the performance results with a substantial feature reduction when utilizing GPU_PDMM, GSDMM, BTM, LFDMM, PTM, and GPU_DMM. For instance, it reduced the feature set size by 79%, 85%, 87%, 85%, 81%, and 88%, respectively, while providing a similar F1 score. Thus, our proposed approach can select features that contribute the most to text classification. Regarding SATM, GPM, and WNTM with TextNetTopics Pro, we get comparable performance with an approximate 1% degradation in F1-score. However, they reduce the feature set size by 81%, 85%, and 88%, respectively.

**FIGURE 8:**
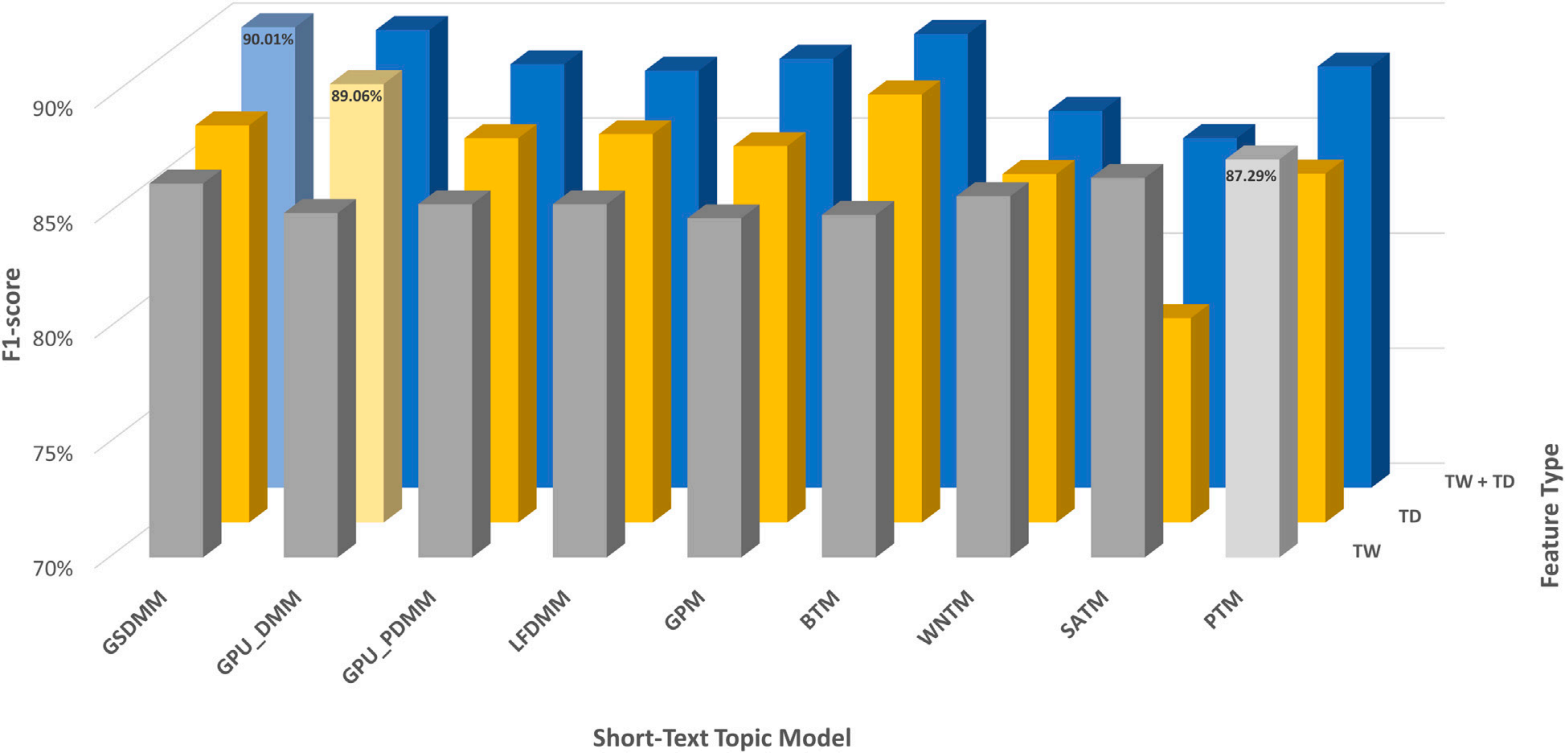
Classification performance on the arXiv dataset when utilizing topical words (TW) extracted by TextNetTopics, topic distribution features (TD) generated by Topic Models, and our proposed approach, combining words of top-ranked topics extracted by TextNetTopics with topic distribution features (TW + TD). The light-colored columns represent the highest achieved values.

In the arXiv dataset, TextNetTopics Pro enhances the F1-score performance of TextNetTopics by 4.93%, 4.84%, 3.90%, 3.76%, 3.05%, 2.77%, 1.01%, and 0.69% when utilizing GPU-DMM, BTM, GPM, GSDMM, GPU_PDMM, LF-DMM, PTM, and WNTM topic models, respectively (as shown in Table 8 and Figure 8). For SATM, interestingly, we got a degradation in performance by 1.30% compared to TextNetTopics, with no significant improvement. In addition, our proposed approach enhances the F1-score using the topic distribution generated with SATM, PTM, GSDMM, GPM, GPU_PDMM, LF-DMM, WNTM, BTM, and GPU-DMM topic models by 6.31%, 3.14%, 2.75%, 2.28%, 1.70%, 1.24%, 1.23%, 1.12%, and 0.83%, respectively.

**FIGURE 9:**
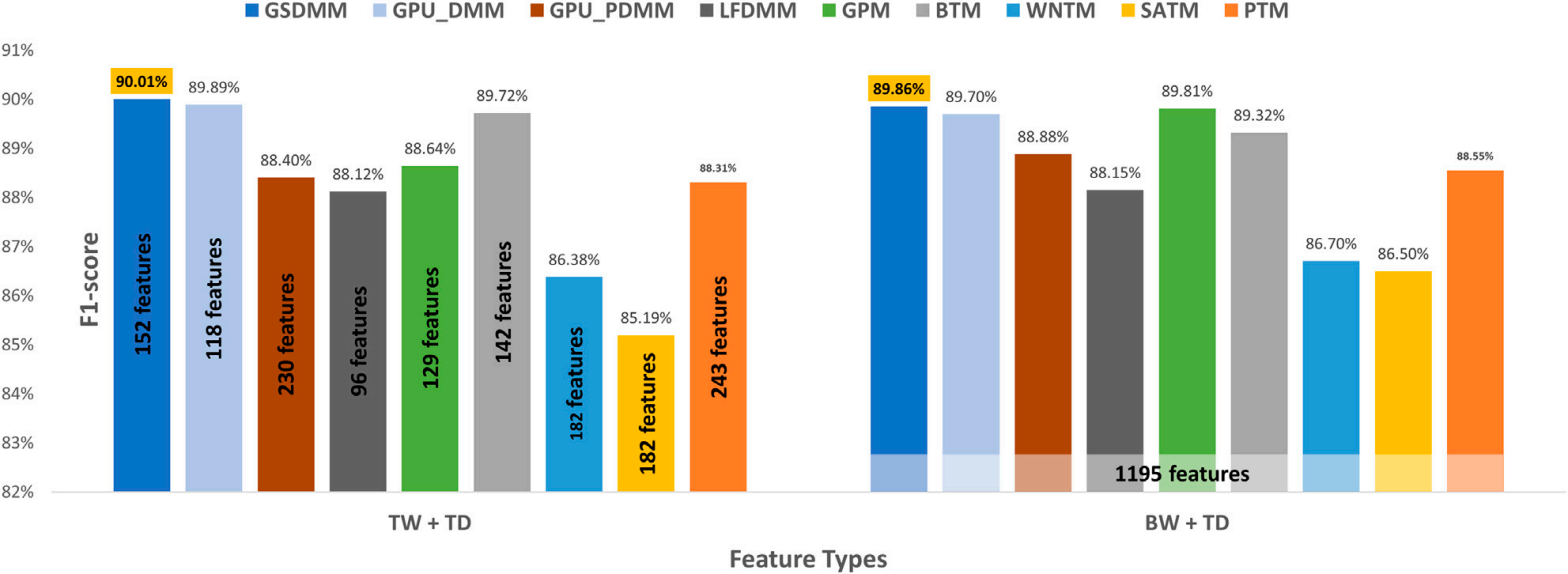
Classification performance on the arXiv dataset when utilizing our proposed approach *versus* taking all preprocessed terms with the semantic features.

Moreover, we compared the performance results obtained when taking all the terms in the preprocessed dataset combined with the semantic features (topic distribution extracted by the topic model) with our enhanced tool (shown in Figure 9). We get similar or a slight improvement in the performance results with a substantial feature reduction by utilizing BTM, GPU-DMM, GSDMM, LF-DMM, PTM, WNTM, and GPU_PDMM with TextNetTopics Pro. For instance, they reduce the feature set size by 88%, 90%, 87%, 92%, 80%, 85%, and 81%, respectively, while providing a comparable F1 score. Thus, our proposed approach can select features that contribute the most to text classification and neglect features that mislead the classifier and degrade its performance. Regarding SATM and GPM with TextNetTopics Pro, we get comparable performance with an approximate 1% degradation in F1-score. However, they reduce the feature set size by 85% and 89%, respectively.

Although the performance of each topic modeling method is dataset dependent, in both datasets, we observe that DMM-based methods (GSDMM and GPU-DMM) and BTM outperform others. Moreover, the SATM and WNTM methods are unable to achieve a high F1 score.

### 6.4 Comparative performance evaluation of TextNetTopics with TextNetTopics Pro on regular-sized text

**FIGURE 10:**
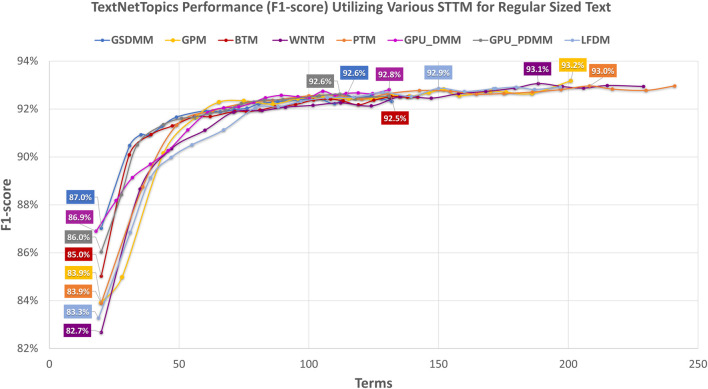
Performance of TextNetTopics over accumulated top-ranked topics using various short-text topic models in the T component on regular-sized text, i.e., titles + abstract (CAMDA dataset). Symbols along the line represent the number of accumulated topics.

**FIGURE 11:**
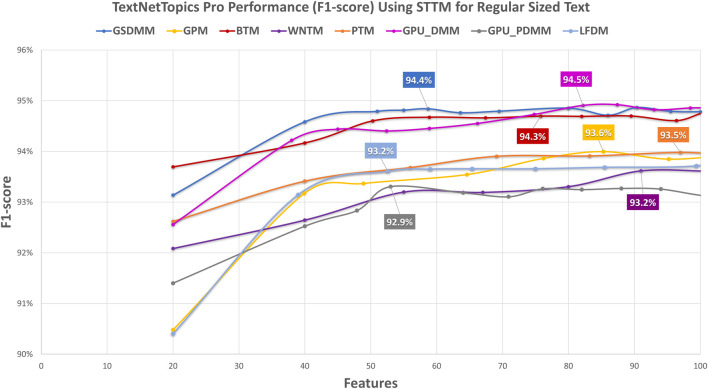
Performance of TextNetTopics Pro over accumulated topic distributions with top-ranked topics using various short-text topic models in the T component on regular-sized text, i.e., titles + abstract (CAMDA dataset). Symbols along the line represent the number of accumulated topics.

In this section, we have comparatively evaluated the performance of TextNetTopics with TextNetTopics Pro on regular-sized text (i.e., title and abstract section) for the CAMDA dataset when short-text topic modeling is employed in the T component. Compared with the use of titles only, Figures 10, 11 imply a significant improvement in F1 score when abstracts are incorporated. This improvement makes sense since incorporating the abstract section provides a more comprehensive and contextually rich representation of the documents, allowing the algorithms to capture a broader range of information and potentially improve their predictive power. For instance, TextNetTopics using GPM, WNTM, and PTM achieved the highest F1 scores, with GPM having 93.2% over the top 20 topics with 201 terms, WNTM obtaining 93.1% over the top 16 topics with 188 terms, and PTM achieving 93.0% over the top 17 topics with 208 terms. Compared with using titles alone, incorporating abstract information resulted in a substantial improvement of 8%, 9%, and 7% for GPM, WNTM, and PTM, respectively. Other topic models showed comparable F1 measures, ranging from 92.5% to 92.9% (as shown in Figure 10). As shown in Figure 11, TextNetTopics Pro showed a remarkable enhancement in the F1-score compared with TextNetTopics. Specifically, TextNetTopics Pro achieved an impressive F1 score of 94.7% with the GPU-DMM topic model. Compared with using titles alone, a substantial improvement (4%) is achieved when incorporating abstract information. GSDMM and BTM showed comparable F1 measures (94.4% and 94.3% respectively). Other topic models showed similar F1 measures, ranging from 93.5% to 93.7%. Tables reporting the title and abstract-based classification performance in the CAMDA dataset utilizing topical words extracted by TextNetTopics, topic distribution features generated by Topic Models, all terms (BOW) combined with topic distribution features, and our proposed approach, which combines words of top-ranked topics extracted by TextNetTopics with topic distribution features, are provided as Supplementary Material.

### 6.5 Performance evaluation of TextNetTopics and TextNetTopics Pro on imbalanced datasets

In our further experiments, we want to test the robustness and effectiveness of our models in handling imbalanced data, particularly in the classification of DILI (Drug-Induced Liver Injury) articles as part of the CAMDA challenge (CAMDA, 2022). After carefully evaluating the performance of various models (as presented in the previous section, section 6.4), in these further experiments, we have focused on the ones that demonstrated the highest performance. Specifically, we chose TextNetTopics with PTM and TextNetTopics Pro with GPU-DMM as the models to be employed for the testing, validation, and comparison.

Table 9 shows that TextNetTopics Pro consistently outperforms the TextNetTopics model across all datasets. It achieves notably higher accuracy, F1-score, precision, and recall measures on most datasets, demonstrating its ability to handle imbalanced data more effectively. For instance, TextNetTopics Pro gains a significant F1-score performance improvement over TextNetTopics with 1.8%, 3.9%, and 14% when applied to T1 till T3 datasets, and 2.3%, 5.0%, 11.5%, and 5.3% when applied to V1 till V4 datasets. Overall, TextNetTopics Pro exhibits superior performance compared with TextNetTopics, establishing itself as being a more robust, reliable, and effective solution for handling imbalanced data in classifying DILI articles.

**TABLE 9:** Performance a) of TextNetTopics, b) of TextNetTopics Pro, c) F1 score improvement achieved by TextNetTopics Pro over TextNetTopics (represented as a percentage %), when applied to CAMDA datasets (T1-T3, V1-V4).

	Accuracy (%)	F1 (%)	Precision (%)	Recall (%)			Accuracy (%)	F1 (%)	Precision (%)	Recall (%)		F1-score improvement
**T1**	86	88	87	90		**T1**	88	90	87	93		+1.8%
**T2**	93	77	68	89		**T2**	95	81	74	89		+3.9%
**T2**	93	46	31	89		**T2**	96	60	46	86		+14.0%
**V1**	86	86	84	89		**V1**	88	89	85	93		+2.3%
**V2**	93	73	61	89		**V2**	95	78	69	89		+5.0%
**V3**	93	47	32	89		**V3**	96	58	45	85		+11.5%
**V4**	86	87	82	92		**V4**	92	92	87	98		+5.3%

## 7 Conclusion

This study evaluated the performance of TextNetTopics on short text using various short-text topic models. TextNetTopics utilizing the PTM topic model reported the highest performance results along both datasets. Moreover, TextNetTopics achieved competing performance metrics with other feature selection algorithms, such as the XGBOOST.

Additionally, in this study, we proposed TextNetTopics Pro, a novel approach that performs feature selection oriented towards the short text classification domain, and presented its application on article title categorization. Our findings show that TextNetTopics Pro improved the overall classification performance of short text by incorporating the two types of representative features learned from the same corpus for semantically richer text representation, topical words, and topic distributions. Among various short-text topic models, TextNetTopics Pro utilizing BTM and DMM-based methods (GSDMM and GPU-DMM), reported the highest performance results along both datasets.

Moreover, our study has demonstrated the robustness and effectiveness of TextNetTopics Pro in handling imbalanced data, particularly in the classification of DILI (Drug-Induced Liver Injury) articles as part of the CAMDA challenge.

The significance of our approach is that it provides dimensionality reduction of the Vector Space Model (VSM) while preserving semantic structures in the text and making sparse short texts more related and topic-oriented. Unlike the BOW model, our approach considers the relationship between words, maintaining semantic and syntactic information in document representation, such as word meanings and context association, and thus alleviating short text sparseness and the low number of feature problems. Such semantically enhanced feature representation enables machine learning algorithms to discover deeper patterns in data and assure better generalization. In addition, it greatly affects the performance of text classifiers in terms of computational time and learning accuracy over the traditional one.

One main limitation of our approach is that the number of topics to detect from the unstructured textual documents is user-defined. However, determining the optimal number of relevant latent topics is not straightforward, as this quantity is dataset dependent and not known in advance. An inadequate or exorbitant number of topics can degrade the predictive performance of the classification algorithms built upon topic modeling. In addition, each topic model requires several other parameters to calibrate, which are critical to their performance, and their configuration is not a tedious task.

## Data Availability

The original contributions presented in the study are included in the article/Supplementary Material, further inquiries can be directed to the corresponding authors.
