# Supplementary figures and images for: TextNetTopics Pro, a topic model-based text classification for short text by integration of semantic and document-topic distribution information
Source: Front Genet. 2023 Oct 5;14:1243874. doi: 10.3389/fgene.2023.1243874 (PMC10585361; doi:10.3389/fgene.2023.1243874)

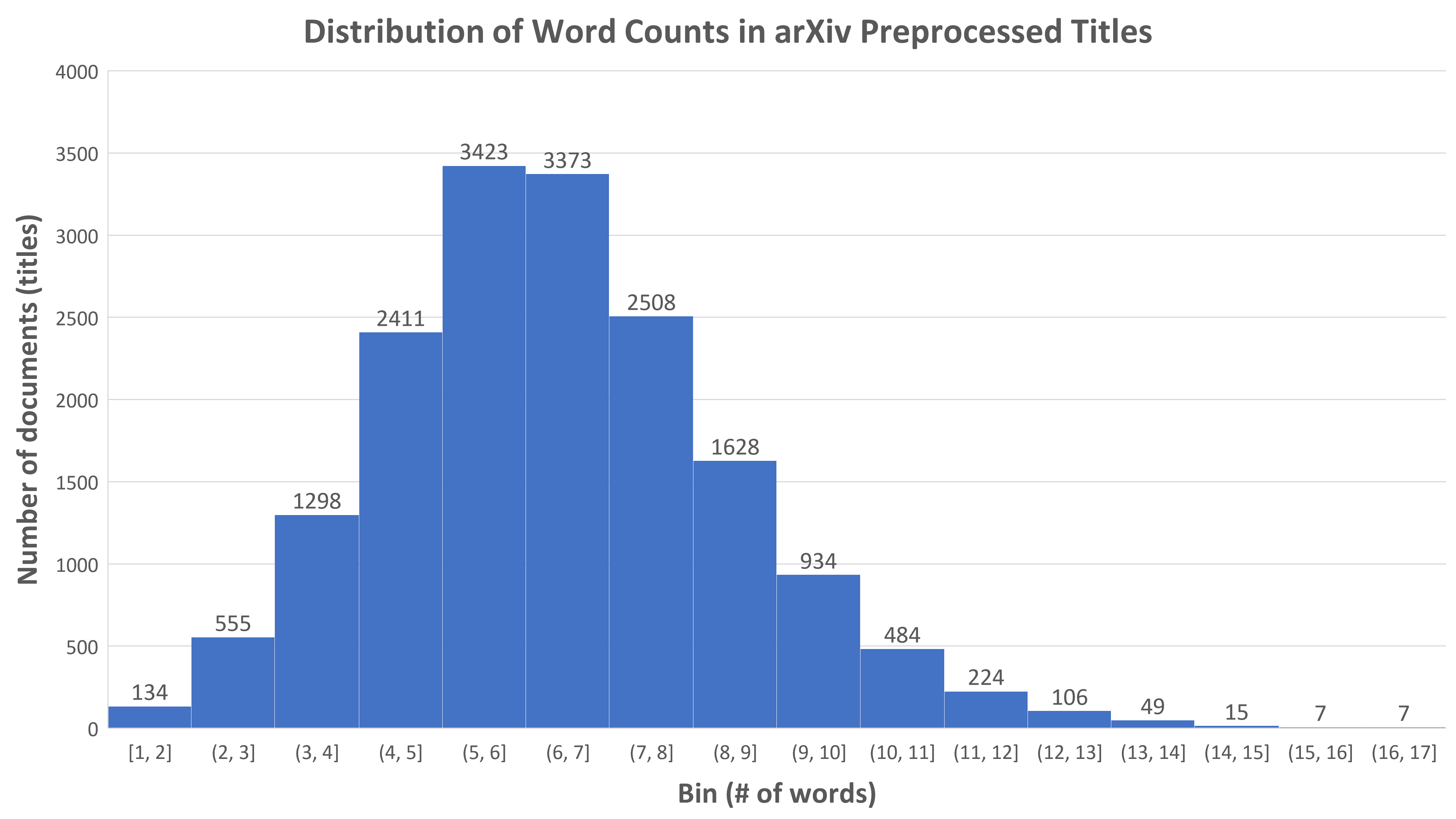

Supplement: Supplementary file 1 [file Image2.tif]

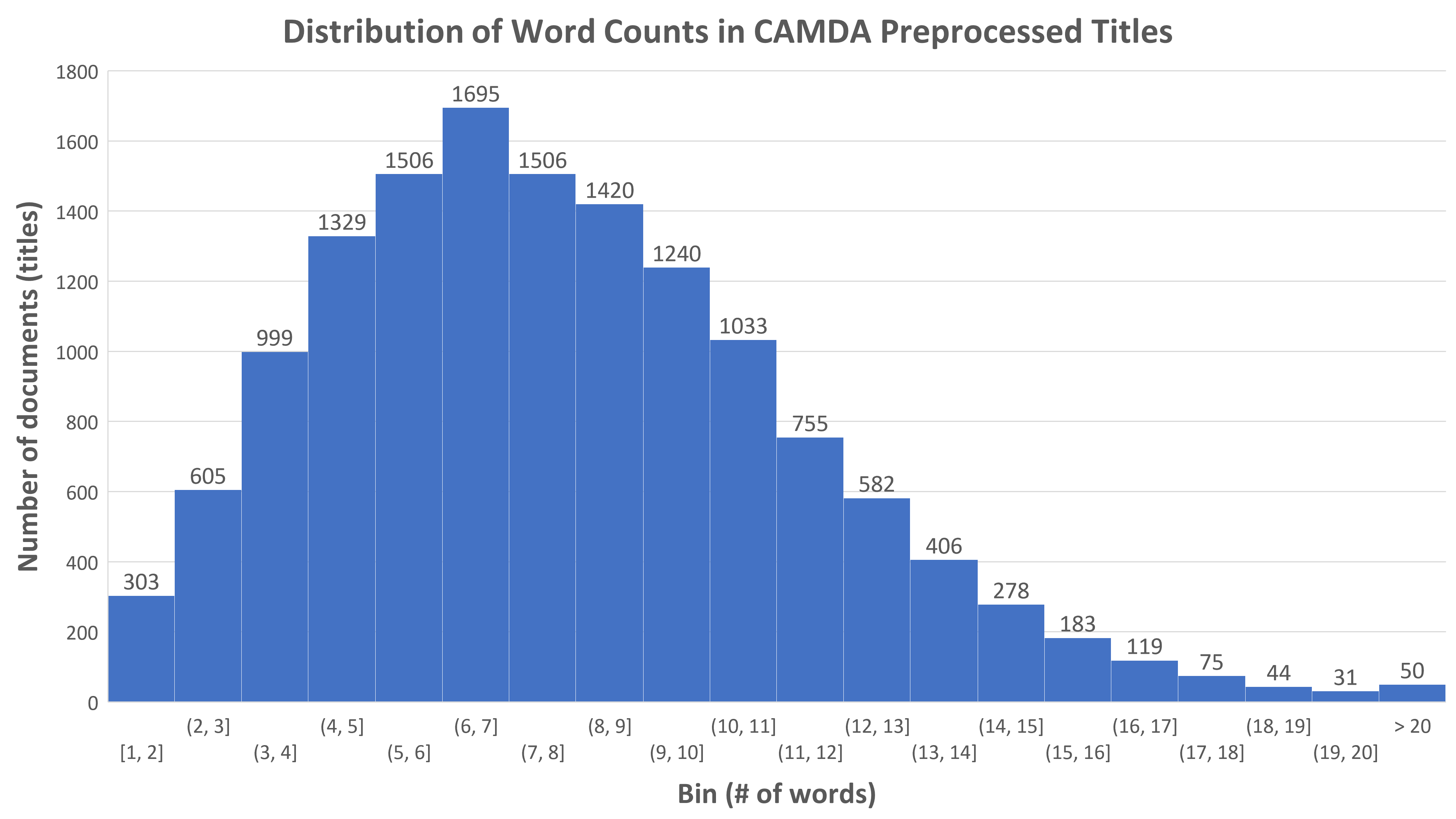

Supplement: Supplementary file 2 [file Image1.tif]
